# The *Tryptophan decarboxylase 1* Gene From *Aegilops variabilis No.1* Regulate the Resistance Against Cereal Cyst Nematode by Altering the Downstream Secondary Metabolite Contents Rather Than Auxin Synthesis

**DOI:** 10.3389/fpls.2018.01297

**Published:** 2018-09-04

**Authors:** Qiulan Huang, Lin Li, Minghui Zheng, Fang Chen, Hai Long, Guangbing Deng, Zhifen Pan, Junjun Liang, Qiao Li, Maoqun Yu, Haili Zhang

**Affiliations:** ^1^Chengdu Institute of Biology, Chinese Academy of Sciences, Chengdu, China; ^2^College of Life Sciences, Sichuan University, Chengdu, China; ^3^University of the Chinese Academy of Sciences, Beijing, China; ^4^School of Basic Medical Sciences, Zunyi Medical University, Zunyi, China

**Keywords:** cereal cyst nematode, *Aegilops variabilis No.1*, tryptophan decarboxylase, secondary metabolite, indole acetic acid

## Abstract

Cereal cyst nematode (CCN, *Heterodera avenae*) is a most important pathogen of wheat and causes tremendous yield loss annually over the world. Since the lack of resistance materials among wheat cultivars, identification and characterization of the resistance-related genes from the relatives of wheat is a necessary and efficient way. As a close relative of wheat with high resistance against CCN, *Aegilops variabilis No.1* is believed to be a valuable source for wheat breeding against this devastating disease. However so far, very few resistance-associated genes have been characterized from this species. In this study, we present that the *tryptophan decarboxylase* genes from *Ae. variabilis No.1* (*AeVTDC1* and *AeVTDC2*) were both induced by CCN juveniles at the early stage of resistance response (30 h post-inoculation), with *AeVTDC1* more sensitive to CCN infection than *AeVTDC2*. Silencing of *AeVTDC1* led to compromised immunity to CCN with more CCN intrusion into roots; while overexpression *AeVTDC1* in *Nicotiana tabacum* dramatically enhanced the resistance of plants by reducing the knots formed on roots. Metabolism analysis showed that the contents of secondary metabolites with activity of resistance to varied pathogens correlated with the expression level of *AeVTDC1* in both *Ae. variabilis No.1* and the transgenic tobacco plants. In addition, the content of IAA was not affected by either silencing or overexpressing of *AeVTDC1*. Hence, our research provided *AeVTDC1* a valuable target that mediates resistance to CCN and root knot nematode (RKN, *Meloidogyne naasi*) without influencing the auxin biosynthesis.

## Introduction

The cereal cyst nematode (CCN, *Heterodera avenae*) is a vital pathogen of graminaceous crops, such as wheat and barley. CCN is widely distributed and causes great production losses (Williamson and Kumar, 2006; Hajihasani et al., 2010; Zheng et al., 2015; Li et al., 2016). Many efforts have been made to identify CCN resistance (Cre) genes. However, gene resource resistant to CCN is scarce in wheat but abundant in its relatives (Montes et al., 2008). *Rha* genes were mapped in barley. Through changing the transcript abundance and composition of cell wall, *Rha2*-mediated CCN resistance drives rapid deterioration of CCN feeding sites (Aditya et al., 2015). *CreX* and *CreY* were identified in *Aegilops* *variabilis* (Barloy et al., 2006). Some resistance lines were bred through hybridization. Although several loci related to CCN resistance have been reported, few of them has been cloned and their biological functions hadn’t been clarified (de Majnik et al., 2003; Safari et al., 2005; Zhang et al., 2016). *Ae. variabilis No.1* (2n = 4x = 28, UUS^v^S^v^), belonging to the genus *Aegilops* of the *Triticeae* tribe, is known as a well-resistant material, which confers strong resistance against CCN and root knot nematode (RKN, *Meloidogyne naasi*) (Barloy et al., 2006; Coriton et al., 2009; Xu et al., 2012; Zheng et al., 2015; Wu et al., 2016).

Plant tryptophan decarboxylase (TDC, 4.1.1.28) catalyzes the formation of tryptamine from tryptophan (Trp) (Giebel and Jackowiak, 1976; Hallard et al., 1997; Canel et al., 1998). Tryptamine is a precursor for the biosynthesis of serotonin, indole alkaloids, and indole acetic acid (IAA) (Bartel, 1997; Valletta et al., 2010; Liu et al., 2012; Dubouzet et al., 2013). Transformation from tryptamine to serotonin is catalyzed by Tryptamine-5-hydroxylase (T5H) (Kang and Back, 2009). TDCs are key enzymes in the biosynthetic pathway of terpenoid indole alkaloids (TIAs), since they link primary to secondary metabolism by converting Trp into tryptamine. Conversion of Trp into tryptamine is a common backbone for many secondary metabolites, which have divergent biological activities regulated by developmental and environmental factors (Mehrotra et al., 2013; Verma et al., 2015). Plant *TDC* cDNA was firstly isolated from *Catharanthus roseus* by screening a cDNA expression library (Hallard et al., 1997; Geerlings et al., 1999). Gradually, a few *TDC* genes had been cloned and characterized from other species, such as *Nicotiana tabacum, Mitragyna speciosa*, and *Withania coagulans* (Berlin et al., 1993; Goddijn et al., 1994; Charoonratana et al., 2013; Jadaun et al., 2017). The biological functions of TDCs have been reported in several plant–pathogen interactions. It was reported that *TDC* gene played a role in resistance against *Malacosoma disstria Hub* and *Manduca sexta L*. through tryptamine, which had adverse effects on their feeding behaviors and physiology (Gill and Ellis, 2006). Serotonin defended against *Magnaporthe oryzae* infection in rice leaves (Gill et al., 2003; Hayashi et al., 2016). Ectopic expression of *TDC1* significantly suppressed the growth of insect pests by sufficient tryptamine accumulation in poplar and tobacco leaf tissue (Gill et al., 2003). The inhibition of TDC enzyme activity with S-αFMT resulted in susceptibility of *Ae. variabilis No.1* to CCN and RKN, which indicated *AeVTDCs* play important roles in resistance to nematodes (Li et al., 2016). However, it remains to be clarified which *AeVTDC* involved in resistance to CCN and RKN and its mechanism of function.

Previous RNA-Seq analysis indicated that the *TDC* genes of *Ae. variabilis No.1* (*AeVTDCs*) showed different expression pattern between control and CCN invaded roots at different time points (Zheng et al., 2015). *AeVTDC1* gene was cloned and its protein had the ability of catalyzing the formation of tryptamine from tryptophan. In this study, we reported *AeVTDC1* played a positive role at the early stage of plant resistance to CCN infection and overexpression of *AeVTDC1* in tobacco led to reduced susceptibility to RKN. Silencing or overexpression of *AeVTDC1* didn’t affect accumulation of IAA, but changed the downstream secondary metabolites of *AeVTDC1*.

## Materials and Methods

### Plant Materials and Culture Condition

Seeds of *Ae. variabilis No.1* and wheat (Fielder) were surface-cleaned by the sterilized water and kept at 4°C for 24 h. Then the seeds were germinated in Petri dishes (5-cm diameter) on wet paper at 20°C under a 16-h light/8-h dark photoperiod. After 2 days, these little seedlings plants were cultured with water or sterilized soil for later use.

*AeVTDC1* transgenic tobacco and wild type (WT) were cultured in the sterilized soil in greenhouse under 25°C and 60% humidity.

### Nematode Hatching, Inoculation, and Staining

Second stage juveniles (J2s) of CCN (*H. avenae*) were hatched with cereal cysts as described previously (Li et al., 2016). For resistance assay and expression inducing assay, about 300 J2s of CCN per pot were inoculated around the root-tips under 19°C. According to the Li’s description, the eggs and second stage juveniles (J2s) of RKN were obtained (Li et al., 2016). For root nematode staining, entire root systems were collected and washed cleanly at 3 days after inoculation (DAI), and immersed in the solution (5% NaClO) for 5 min. Then roots were soaked in tap water for 15 min to remove residual NaClO. The roots were rinsed in boiling stain solution (acid fuchsin, 0.5 g/L) for 30 s, washed with tap water, then placed in 30 mL glycerin acidified with few drops of 5 mol/L HCl for water bath heating 30 s. The dyed roots were kept in glycerin for storage at 4°C. The number of visible pink-stained nematodes present within the roots was counted under a light microscope (Leica, DM3000 LED). At least nine replicate samples (individual plants) were counted for each treatment (Aditya et al., 2015).

### RNA Extraction, Reverse Transcription, and Quantitative Real-Time PCR (QPCR)

To detect gene expression after CCN infection, two-leaf stage seedlings were cultured in sterilized soil (four seedlings each pot), and inoculated with nematodes (300 J2s/pot). The root tissues were respectively collected at 0, 30 h and 3, 9 days. All these samples were stored at -80°C for RNA extraction.

Total RNA was extracted using the TRIzol-A^+^ reagent (Tiangen, Beijing, China) according to the manufacturer’s instructions. The cDNA synthesis was carried out using the ReverTra Ace qPCR RT Kit (TOYOBO). QPCR was conducted as described (Wang et al., 2013). Gene-specific primers were designed to amplify PCR products about 100–200 bp, and listed in **Supplementary Table 1**. *Elongation factor1-α* (*EF1α*) mRNA was employed as an internal control for normalization (Livak and Schmittgen, 2001). Each sample or treatment was tested in three biological repeats and experiment was performed for three times. The differences were analyzed by *t*-test and data were presented by software Origin 8.6.

### Barley Stripe Mosaic Virus (BSMV)-Mediated Gene Silencing

The plasmids used for gene silencing were constructed as previously described (Holzberg et al., 2002). 751∼951 bp of *AeVTDC1* coding sequence (ORF) was cloned and ligated to BSMV γ plasmid through NheI to construct plasmid for silencing *AeVTDC1*. Primers used were listed in **Supplementary Table 1**.

Infectious BSMV RNA was prepared from each linearized plasmid (α and γ digested with MluI, β digested with SpeI) by *in vitro* transcription using a Large Scale RNA Production System (T7 RiboMAXTM Express Large Scale RNA Production System). The BSMV inoculum was made by combining an equimolar ratio of α, β, and γ transcripts with excess inoculation buffer containing a wounding agent (GKP buffer: 50 mM glycine, 30 mM dipotassium phosphate, pH 9.2, 1% bentonite, 1% celite) as previously described (Holzberg et al., 2002). The second leaves of two-leaf seedlings were inoculated with BSMV inoculum. BSMV γ empty vector were used as negative controls.

Barley stripe mosaic virus-treated plants were kept in a cultivation chamber at 25°C with 60% humidity. When the virus phenotype was observed (about 10 days after BSMV inoculation), new roots of these plants were sampled and used for RNA isolation. The silencing efficiency for the target gene and expression levels of other related genes compared with control were examined by QPCR. The primers for QPCR were listed in **Supplementary Table 1**.

### Overexpression of *AeVTDC1* in Tobacco and RKN Resistance Assay

PCAMBIA1300-based T-DNA vector was chosen as the skeleton and hygromycin was replaced with bar gene. CaMV35S promoter and NOS terminator were amplified using pJG045 as a template to drive and terminate gene expression (Zhao et al., 2013). For generation of the overexpression construct, the ORF of *AeVTDC1* was PCR amplified using template with primers (**Supplementary Table 1**) (Li et al., 2016). *AeVTDC1* ORF was fused to N terminal of yellow fluorescent protein (YFP) sequence and together inserted into the modified binary vector to express *AeVTDC1*-YFP fusion protein. The insertion sequences were confirmed by nucleotide sequencing. Overexpression construct was introduced into *Agrobacterium tumefaciens* strain EHA105 for tobacco transformation (Horsch et al., 1985). Cultivar tobacco (Mammoth Gold) was used for transformation. Positive transformants and their offsprings were screened by PCR with specific primers of *AeVTDC1*. Stable lines (L120 and L133) were selected for QPCR analysis of expression of genes and RKN resistance assay.

In resistance assay, L120, L133 and WT seeds were simultaneously germinated and cultured with sterilized soil under 25°C. Six-leaf seedlings were transplanted into soil containing RKN. The root knots were calculated and analyzed after 8 weeks. No less than 15 individuals each line were used for counting. The photographs were taken by camera (Canon).

### IAA Quantitative Analysis

The IAA in the roots were measured by UPLC–MS/MS as previously described (Fu et al., 2012).

### Isolation of Secondary Metabolites and Analysis by UPLC–MS/MS

All fresh roots were collected and freeze-dried for metabolites profiling. Three replicates were used in each treatment. The method was slightly modified according to the description (Chen et al., 2013). Each 0.1 g sample was powdered in liquid nitrogen using a mortar and pestle before 1 mL of extraction solution (70% aqueous methanol) was added, and the mixture was stored overnight in the dark at 4°C. The mixture was then centrifuged at 4°C at 10,000 *g* for 10 min, and each supernatant was filtered through a 0.22-μm Millipore filter before HPLC–MS/MS analysis.

#### HPLC Conditions

The sample extracts were analyzed using an LC–ESI–MS/MS system (HPLC, Shim-pack UFLC SHIMADZU CBM30A system^1^; MS, Applied Biosystems 6500 Q TRAP ^2^). The analytical conditions were as follows, HPLC: column, Waters ACQUITY UPLC HSS T3 C18 (1.8 μm, 2.1 mm × 100 mm); solvent system, water (0.04% acetic acid): acetonitrile (0.04% acetic acid); gradient program, 100:0 V/V at 0 min, 5:95 V/V at 11.0 min, 5:95 V/V at 12.0 min, 95:5 V/V at 12.1 min, 95:5 V/V at 15.0 min; flow rate, 0.4 ml /min; temperature, 40°C; injection volume: 2 μl. The effluent was alternatively connected to an ESI-triple quadrupole-linear ion trap (Q TRAP)–MS.

#### ESI–Q TRAP–MS/MS

LIT and triple quadrupole (QQQ) scans were acquired on a triple quadrupole-linear ion trap mass spectrometer (Q TRAP), API 6500 Q TRAP LC/MS/MS System, equipped with an ESI Turbo Ion-Spray interface, operating in a positive ion mode and controlled by Analyst 1.6 software (AB Sciex). The ESI source operation parameters were as follows: ion source, turbo spray; source temperature 500°C; ion spray voltage (IS) 5500 V; ion source gas I (GSI), gas II (GSII), curtain gas (CUR) were set at 55, 60, and 25.0 psi, respectively; the collision gas (CAD) was high. Instrument tuning and mass calibration were performed with 10 and 100 μmol/L polypropylene glycol solutions in QQQ and LIT modes, respectively. QQQ scans were acquired as MRM experiments with collision gas (nitrogen) set to 5 psi. DP and CE for individual MRM transitions were done with further DP and CE optimization. A specific set of MRM transitions were monitored for each period according to the metabolites eluted within this period.

## Results

### Expression of *AeVTDC1* Was Strongly Induced by CCN Infection

**FIGURE 1:**
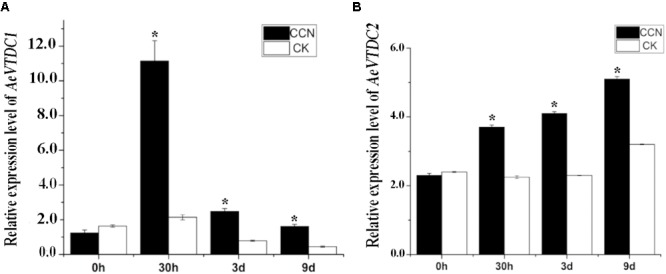
Expression of *AeVTDC1* and *AeVTDC2* gene after CCN inoculation in *Aegilops variabilis No.1* roots. Two-leaf stage seedlings were used for CCN J2 inoculation or mock inoculation. CCN treatment group was inoculated with nematodes (300/pot, 4 seedlings/pot), and the control group was inoculated with same volume water. The roots tissues were respectively collected at 0, 30 h and 3, 9 days post-inoculation for RNA extraction and QPCR. The relative expressions were normalized with the *AeVEF1α*. Data represent the mean ± standard deviation of three replicate samples. The asterisk above the bars indicate values that are significantly different (*P* < 0.05).

Previous RNA-seq results indicated transcripts of several *AeVTDCs* were much higher in CCN-treated roots than that in non-treated roots. And primary results revealed inhibition of AeVTDC enzyme activity suppressed resistance to CCN (Li et al., 2016). To determine which member of *AeVTDC* family mainly contribute to CCN resistance, expression pattern of *AeVTDC1* and *AeVTDC2* were tested and compared at 0 hour (h), 30 h, 3 day (d), 9 day post-inoculation (dpi) of CCN. At 0 hpi, the expression level of the *AeVTDC1* and *AeVTDC2* were similar in the CCN-treated and control roots. At 30 hpi, the expression of *AeVTDC1* was strikingly induced in CCN-treated roots, which is almost sixfold of that in control sample. Expression of *AeVTDC2* was also induced and about twofold to that in control root. At 3 and 9 dpi, expressions of *AeVTDC1* remained induced and were nearly threefold of that in control sample. And expressions of *AeVTDC2* were still higher but less than two times of that in control sample (**Figure 1**). These results indicated that expression of *AeVTDC1* was much more sensitive to CCN infection than *AeVTDC2*. Hence, *AeVTDC1* was chosen as the candidate gene for the resistance assay.

### Silencing of *AeVTDC1* Compromised Resistance to CCN in *Ae. variabilis No.1*

**FIGURE 2:**
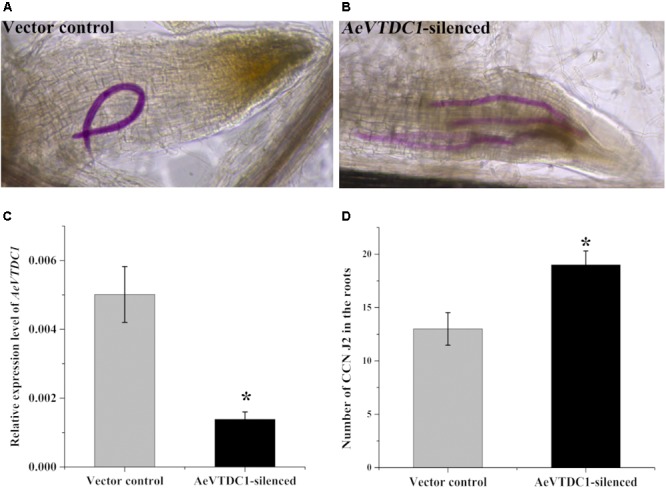
Silencing of *AeVTDC1* gene compromised the resistance to CCN infection in *Ae. variabilis No.1*. **(A,B)** Roots were stained with acid fuchsin to dye nematodes. The visible pink nematodes were counted and photographed. **(A)** One vision of vector control root under microscope; **(B)** one vision of the *AeVTDC1*-silenced root. **(C)** The relative expression of *AeVTDC1* in roots of vector control and *AeVTDC1*-silenced plants. VIGS inoculation was operated at the two-leaf stage of plant. About 2 weeks after inoculation, the roots of plants displayed BSMV infection symptoms were individually collected to affirm the VIGS effect by QPCR. The results were normalized with the *AeVEF1α*. **(D)** Number of CCN in *AeVTDC1*-silenced roots and vector control plants. The successful silencing plants were used for CCN J2 inoculation. Roots were collected and dyed with acid fuchsin 3 days after CCN inoculation. The number of visible pink-stained nematodes present within the roots was counted under a light microscope. The data were means ± SE. No less than 15 plants were used and calculated in each treatment. The asterisk represented significant differences (*P* < 0.05).

Virus induced gene silencing is an efficient and fast technology in gene function analysis (Holzberg et al., 2002; Liu et al., 2002). BSMV based silencing system has been widely applied in monocots (Pacak et al., 2010). In our study, we found the expression of *AeVTDC1* was strikingly induced at 30 hpi, which is almost sixfold of that in control sample. To directly investigate whether *AeVTDC1* gene participates in resistance to CCN in *Ae. variabilis No.1*, we utilized BSMV-mediated gene silencing to knock down expression of *AeVTDC1* in roots and observed CCN infection. Expression of *AeVTDC1* in roots of *AeVTDC1*-silenced plants was about 30% of that in vector control (**Figure 2C**), which indicated VIGS worked well in the roots. Furthermore, the expression of *AeVTDC2* in roots of *AeVTDC1*-silenced plants was similar with that in vector control (**Supplementary Figure 1**). The number of dyed CCN J2 in the roots of *AeVTDC1*-silenced plants increased more than 45% compared with that of vector control plants at 3 dpi (**Figure 2D**). The data was collected from at least nine replicates each time, and the experiment was operated three times. This result showed silencing *AeVTDC1* in *Ae. variabilis No.1* plants compromised resistance to CCN at the early stage and indicated *AeVTDC1* regulates early immune responses to CCN.

### Silencing of *AeVTDC1* Changed the Contents of Secondary Metabolites and Expression of Related Genes

To analyze in more details of how *AeVTDC1* regulated resistance to CCN in *Ae. variabilis No.1*, the changes of secondary metabolite and expression of related genes were detected in the roots of *AeVTDC1*-silenced plants.

**FIGURE 3:**
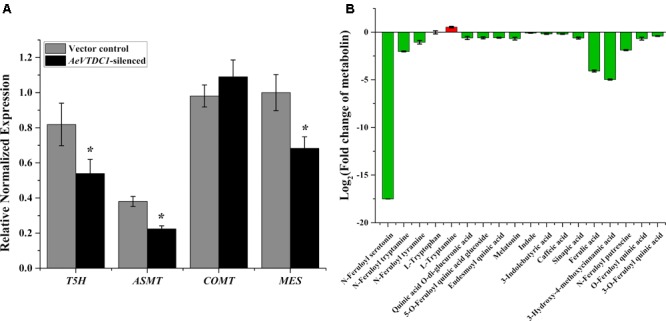
Silencing of *AeVTDC1* affected expression of downstream genes and contents of relative secondary metabolites in *Ae. variabilis No.1* plants. **(A)** Relative expressions of *AeVTDC1* downstream genes (*T5H, ASMT, COMT*, and *MES*) were detected in *AeVTDC1*-silenced and vector control roots. **(B)** Fold changes of secondary metabolites in *AeVTDC1*-silenced compared to that in vector control roots. The content of secondary metabolite was detected by UPLC-ESI-MS/MS. Data represent the mean ± standard deviation of three replicate samples. Asterisk above the bars indicate values that were significantly different (*P* < 0.05).

Specific primers were designed to analyze the expression of genes downstream of *TDC*. Tryptamine-5-hydroxylase (T5H) catalyzes tryptamine transform into serotonin. Acetylserotonin *O*-methyltransferase (ASMT) catalyzes the synthesis of melatonin (Back et al., 2016). Methyl easterase (MES) is a key gene in the synthesis pathway of indole alkaloids. In the *AeVTDC1*-silenced plant roots, the expression of *T5H, ASMT*, and *MES* gene markedly declined comparing with vector control (**Figure 3A**). However, the content of tryptamine and melatonin had no significant changes in the *AeVTDC1*-silenced plant roots, and the content of serotonin might be too low to be detected both in *AeVTDC1*-silenced and vector control plants (**Figure 3B**). Indole and 3-Indolebutyric acid content slightly reduced. Caffeic acid 3-*O*-methyltransferase (COMT) methylates caffeic acid and 5-hydroxyferulic acid respectively to form ferulic acid and sinapic acid (Doorsselaere et al., 2010; Back et al., 2016). QPCR results displayed that the expression of *COMT* gene was unchanged (**Figure 3A**), but the content of caffeic acid, ferulic acid, and sinapic acid obviously decreased in the roots of *AeVTDC1*-silenced plants compared with vector control plants. Furthermore, the derivatives of serotonin, derivatives of tryptamine, and ferulic acid derivatives sharply declined in *AeVTDC1*-silenced plants. N-Feruloylserotonin went down 17 times. *N*-Feruloyltryptamine, *N*-Feruloylputrescine, and 3-*O*-Feruloylquinic acid also had significant reduction (**Figure 3B**). The results showed that silencing of *AeVTDC1* gene altered the profile of downstream metabolin. The changes of metabolite (tryptamine derivatives, serotonin derivatives, ferulic acid and its derivatives) might be an important aspect for *AeVTDC1* to regulate resistance to CCN in *Ae. variabilis No.1*.

### Accumulation of IAA, Transcripts of Its Biosynthetic and Signaling Genes Were Unaffected by Silencing of *AeVTDC1*

TDC catalyzes conversion from tryptophan to tryptamine, and tryptamine is a precursor for the biosynthesis of IAA (Dubouzet et al., 2013). Except for regulation of plant development, IAA also involves in plant defense to biotic stress and abiotic stress (Mathesius, 2010; Navarro, 2016).

**FIGURE 4:**
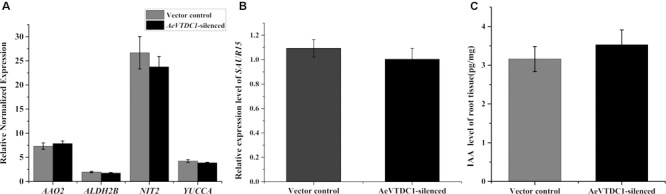
Expression of IAA biosynthetic and signaling genes and IAA contents in roots of *AeVTDC1*-silenced and control plants. **(A)** Relative expression of IAA biosynthetic genes *AAO2, ALDNH2B, NIT2, YUCCA* were detected in *AeVTDC1*-silenced and vector control roots. **(B)** Expression of IAA signaling gene *SAUR15* in *AeVTDC1*-silenced and vector control roots. The results were normalized with the *AeVEF1α*. **(C)** Level of IAA in *AeVTDC1*-silenced and vector control roots. The content of IAA was detected by UPLC–MS. Data represent the mean ± standard deviation of three replicate samples.

To understand whether IAA biosynthesis and signaling pathway were altered, we analyzed the transcripts changes of biosynthetic genes and signaling genes of IAA when *AeVTDC1* was silenced. QPCR results showed there were no significant differences in the expression levels of biosynthesis genes (indole-3-acetaldehyde oxidase, *AAO2*; indole-3-pyruvate monooxygenase, *YUCCA*; nitrilase 2, *NIT2*; aldehyde dehydrogenase, *ALDH2B*) and signaling genes (small auxin-upregulated RNA, *SAUR15*) in *AeVTDC1*-silenced roots and control vector roots (**Figures 4A,B**). Liquid chromatography–mass spectrometry (LC–MS) data further attested the level of IAA in the roots of *AeVTDC1*-silenced plants was similar with that in vector control plants (**Figure 4C**). These results demonstrated silencing of *AeVTDC1* had no effect on expression of biosynthetic and signaling genes of IAA and IAA accumulation in *Ae. variabilis* plants. There might be other factors related to the resistance reduction in the *AeVTDC1*-silencing plants.

### Overexpression of *AeVTDC1* in Tobacco Improved Resistance to RKN

**FIGURE 5:**
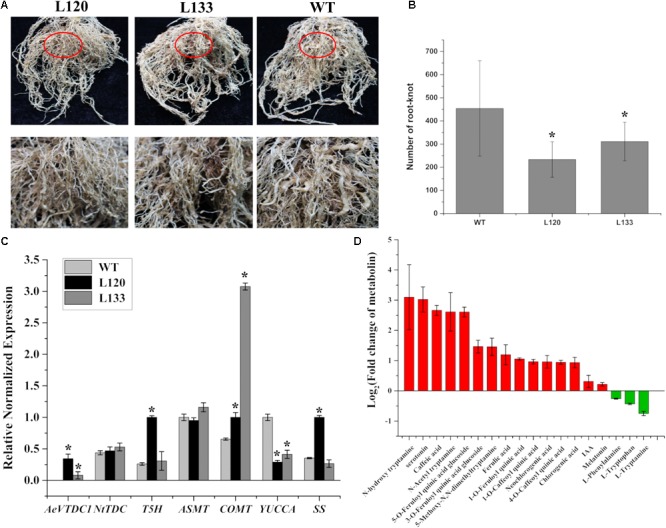
Number of root knots formed on roots of *AeVTDC1* overexpressing tobacco plants and non-transgenic plants, and contents of downstream metabolites in roots were respectively analyzed. **(A)** Phenotypes of the root tissues of non-transgenic and *AeVTDC1* transgenic tobacco. L120 and L133 were two independent lines of *AeVTDC1* transgenic tobacco. The first row showed the overall view of root tissues, and the second row displayed the partial tissue circled with red oval in the first row. Photos were taken 2 months after planting in soil containing RKN. **(B)** Statistic data of root knots numbers formed on *AeVTDC1* overexpressing tobacco and non-transgenic tobaccos. The mean number of knots was calculated and presented. More than 15 replicates were used for counting. **(C)** Relative expressions of *AeVTDC1, NtTDC, TDC* downstream genes *T5H, ASMT, COMT, SS* in tobacco, and IAA biosynthetic related genes *YUCCA* were detected in root tissues of L120, L133, and WT. **(D)** Fold change of secondary metabolite in *AeVTDC1* overexpressing tobacco plants (L120) and WT roots. The content of secondary metabolite was detected by UPLC-ESI-MS/MS. The error bar represented the standard error. ^∗^*P*-value < 0.05.

To test whether *AeVTDC1* involved resistance to other pathogen, *AeVTDC1* was overexpressed in tobacco. Stable *AeVTDC1* transgenic tobacco plants (L120 and L133) were obtained to monitor the susceptibility to RKN. *AeVTDC1* transgenic plants and WT plants with similar growth state were planted in the homogenous soil containing RKN. Two months later, all root tissues of plants were taken out to count the number of root knots and photographed (**Figure 5A**). The knots formed on transgenic plants were much smaller than that on WT. Statistical data revealed that the number of knots formed on the transgenic plants was much less than that on WT (**Figure 5B**). The results demonstrated ectopic expression of *AeVTDC1* in tobacco enhanced defense to RKN and led to reduction of root knots.

To further analyze which downstream products of TDC improved resistance to RKN in transgenic tobacco, the roots of the most resistant line (L120) and WT were respectively collected for basic metabolite profile and related gene expression analysis. We found that the expression of *NtTDC* was similar between L120 and WT (**Figure 5C**). And downstream genes and substances in tryptophan metabolism were also detected. The content of tryptamine showed no difference in the transgenic tobacco comparative with WT (**Figure 5D**). The tryptamine may be easily changed to tryptamine derivatives (*N*-hydroxytryptamine, *N*-Acetyltryptamine, and 5-Methoxy-*N,N*-dimethyltryptamine) in the tobacco, which showed obvious increase in L120. Serotonin accumulation strikingly increased in the roots of L120, almost eightfold greater than that of WT plants (**Figure 5D**), and expression of *T5H* was also obviously upregulated in the roots of L120 (**Figure 5C**). The content of IAA and melatonin had no obvious difference (**Figure 5D**), and the biosynthesis gene *ASMT* of melatonin similarly had no change, while IAA biosynthesis gene *YUCCA* was down-regulated compared to the WT (**Figure 5C**). Moreover, we discovered that the expression of *COMT* and the content of ferulic acid and ferulic acid derivatives (1-*O*-Feruloylquinic acid, 3-*O*-Feruloylquinic acid glucoside, 5-*O*-Feruloylquinic acid glucoside) were higher in transgenic tobacco than in WT (**Figures 5C,D**). Furthermore, we also found the level of strictosidine synthase (SS) expression was improved in *AeVTDC1* in tobacco. The contents of caffeic acid, chlorogenic acid and some quinine (such as 1-*O*-Caffeoylquinic acid, 4-*O*-Caffeoylquinic acid) increased by overexpression *AeVTDC1* in tobacco (**Figure 5D**). These results revealed that the content changes of metabolite (serotonin, tryptamine derivatives, ferulic acid and its derivatives) in transgenic tobacco might contribute to the enhanced resistance to RKN in tobacco. Moreover, the related genes expressions were also detected in L133. Expression of *AeVTDC1* was higher in L120 than that in L133. Expression of *NtTDC, COMT, ASMT*, and *YUCCA* was similar in the roots of L133 and L120. However, Expression of *T5H* and *SS* showed no obvious difference in the roots of L133 and WT (**Figure 5C**). The expression differences might account for the different resistances by *AeVTDC1* overexpression.

## Discussion

### The Role of AeVTDC1 in Resistance Against CCN and RKN

CCN (*H. avenae*) is soil-borne and invades plants from roots. Production losses caused by CCN gradually become bigger in recent years. However, it is difficult to control CCN disease because CCN infection is not easy to be observed. Bringing resistance-related genes from relatives into wheat has been known as an efficient strategy to enhance wheat resistance to CCN. However, few resistance-related genes were identified. High-throughput methods like RNA-seq and microarray analysis are widely used to find differential expression genes, which provide a mass of candidate genes for following work.

In our previous work, RNA-sequencing was operated to find out differential expression genes before and after CCN infection in *Ae. variabilis No.1* (Xu et al., 2012). There were a large number of differential expression genes at 30 hpi, when the early response was conferred to CCN infection. Whereas there were fewer differential expression genes at 3 and 9 dpi, when CCN J2 had migrated in vascular tissues and developed into J2∼J3 stage. It was reported that most of differential expression genes gathered at 3 and 8 dpi in RNA-sequencing analysis of incompatible wheat and a compatible control cultivar infected with *H. avenae* at 24, 3, and 8 dpi (Kong et al., 2015). The different responses might indicate a different resistance mechanism in *Ae. variabilis No.1*.

Previous analysis showed transcripts of several genes in tryptophan metabolism were induced by CCN, and *AeVTDCs* as the key gene were also induced at the 30 hpi early response. In this study, *AeVTDC1* and *AeVTDC2* were further verified to be induced by CCN at 30 hpi, which was accordant with the transcriptome data (Xu et al., 2012).

In the wheat genome, there are more than 15 copies of *TaTDCs* which showed various tissue expression patterns (Choulet et al., 2014). Expression patterns of two *TaTDCs* (*TaTDC1* and *TaTDC2*), highly expressed in roots and with highest homology to *AeVTDC1*, were tested after CCN inoculation. Expression of *TaTDC1* was induced after CCN inoculation, while expression of *TaTDC2* showed no obvious changes after CCN infection (**Supplementary Figure 2**). The different expression pattern of the various isoforms might indicate that not all *TaTDCs* isoforms respond to CCN. Biological functions of several plant *TDCs* were gradually disclosed. Overexpression the *Catharanthus roseus TDC* gene in plants (tobacco, poplar, canola, *Petunia hybrida*) gave rise to tryptamine accumulation in transgenic plants, which improved tobacco resistance to *Manduca sexta* and poplar resistance to *Malacosoma disstria* (Thomas et al., 1999; Leech et al., 2000; Gill et al., 2003). In *Ae. variabilis No.1* genomes, there were at least three *TDC* genes that may have different functions in downstream metabolism process from tryptamine (Facchini et al., 2000; Byeon et al., 2014). However, which *AeVTDC* participated in CCN resistance remained unclear. In this study, expression of *AeVTDC1* showed greater changes than *AeVTDC2* after CCN infection (**Figures 1A,B**). Here, the role of *AeVTDC1* in CCN resistance was directly studied. Silencing of *AeVTDC1* weakened resistance to CCN and led more CCN invading into roots (**Figure 2**). This indicated the positive regulation of *AeVTDC1* in resistance to CCN. It needs further study whether there was function redundancy among *AeVTDC1* and its homologs. Moreover, the role of *AeVTDC1* in RKN resistance was also tested by overexpression in tobacco. *AeVTDC1* overexpression reduced knots on roots and it revealed *AeVTDC1* plays a positive role in RKN resistance as in CCN resistance (**Figures 5A,B**). The different resistance on L120 and L132 might be related to the expression level of *AeVTDC1* in tobacco (Zhang et al., 2013). Hence, we suggested the broad spectrum resistance of *AeVTDC1* to crucial nematodes.

### Relationships Between Downstream Secondary Metabolites of *AeVTDC1* and Nematode Defense

In the plant kingdom, diverse metabolites derived from Trp are found and play an important role in the plant immunity and rice (Bohlmann et al., 1995; Zhao and Last, 1996; Matsukawa et al., 2002; Kang and Back, 2009; Ueno et al., 2010; Ishihara et al., 2011; Dharmawardhana, 2013; Dubouzet et al., 2013; Hayashi et al., 2016; Lu et al., 2018). Metabolites, liking tryptamine, serotonin or their derivatives, have strong antioxidant activities and regulate resistance to avoid damage from pathogen attacks (Kang and Back, 2009; Dubouzet et al., 2013). The dramatic increased serotonin suppress leaf damage outside the halo, block expansion of the browning area and attenuate symptom of plant growth inhibition (Hayashi et al., 2016). Ectopic expression of *Camptotheca acuminata TDC1* gene allowed sufficient tryptamine to accumulate in poplar and tobacco leaf tissue to significantly suppress the growth of insect pests (Gill et al., 2003). Melatonin-rich rice plants exhibit resistance to herbicide-induced oxidative stress (Park et al., 2013). Recently, it was reported that *CYP71A1* mutants with less serotonin content were more susceptible to rice blast *Magnaporthe grisea*, but more resistant to rice brown spot disease *Bipolaris oryzae1*, rice brown planthopper and striped stem borer (Lu et al., 2018).

In this study, serotonin concentration in *AeVTDC1* transgenic tobacco roots was eightfold greater than in roots from WT plants, while tryptamine and melatonin remained unchanged (**Figure 5D**). No change of tryptamine was reasonable since it might be easily transformed to its derivatives (*N*-hydroxytryptamine, *N*-Acetyltryptamine, and 5-Methoxy*-N,N*-dimethyltryptamine), which increased in the transgenic tobacco. Our results were similar to that in overexpression of *Catharanthus TDC* in cell cultures of *Peganum harmala* (Leech et al., 2000). Serotonin and tryptamine derivatives, caffeic acid, chlorogenic acid and several quinines remarkably increased in transgenic tobacco (**Figure 5D**). Except for function in plant immunity, serotonin, feruloylserotonin, and 4-coumaroylserotonin were reported contributed to delay senescence of rice (Kang and Back, 2009). High level of serotonin accumulation in rice caused stunt phenotype (Kanjanaphachoat et al., 2012). Even though elevated level of serotonin accumulated in *AeVTDC1* transgenic tobacco, the plants didn’t show stunted growth (not shown) as overexpression of *TDC* in rice (Kanjanaphachoat et al., 2012). In the VIGS assay, the serotonin wasn’t either detected in *AeVTDC1*-silencing root or in the control roots. That’s probably because of too low level of serotonin in roots of *Ae. variabilis No.1*. Contents of *N*-Feruloylserotonin and *N*-Feruloyltryptamine markedly decreased in the *AeVTDC1*-silencing plants comparing with the control (**Figure 4B**). Ferulic acid is a well-known phytoalexins, which inhibited the growth of necrotrophic bacteria *Dickeya dadantii* (Pérez-Bueno et al., 2016). Furthermore, the decrease of soluble free and soluble conjugated phenolic acids, such as soluble hydrolyzable ferulic acid and sinapic acid, reduced the attraction of *Diabrotica virgifera virgifera* to the root of maize (Erb et al., 2015). In our study, ferulic acid significantly increased in the *AeVTDC1* transgenic tobacco, and decreased in *AeVTDC1*-silencing plants (**Figures 4B, 5D**). Here, we found altered expression of *AeVTDC1* changed secondary metabolites and nematode resistance. It still needs further studies of direct effects of serotonin, ferulic acid, indole alkaloids, quinines and their derivatives on nematodes.

### Regulation of IAA Biosynthesis by *AeVTDC1* and the Relationship Between IAA and CCN Resistance

Plant hormone IAA biosynthesis has several main pathways from Trp, respectively catalyzed by TDC, YUCCA, and NIT (Sugawara et al., 2009; Zhao, 2010). It is well-known that auxin always regulates plant defense as a negative regulator in plant immune system (Mathesius, 2010; Navarro, 2016). A series of evidence has demonstrated that auxin plays roles in balancing plant defense responses and growth in plants. Lionel Navarro reported the repression of auxin signaling made for the improving of bacterial resistance in Arabidopsis (Navarro et al., 2006). Overexpression of *OsGH3.1* and *OsGH3.8* in rice reduced the IAA content, influence cell growth, and enhanced disease resistance to both fungal and bacterial pathogens (Domingo et al., 2009). In this work, the content of IAA was not changed by silencing *AeVTDC1* in *Ae. variabilis No.1* (**Figure 3C**). There was also no change about the content of IAA in the *AeVTDC1* transgenic tobacco comparing with WT (**Figure 5D**). Silencing or overexpression of *AeVTDC1* gene also had no effects on the expression of IAA biosynthesis and signaling genes (**Figures 3A,B, 5C**). The results indicate *AeVTDC1* might not function to regulate IAA biosynthesis. Moreover, we used two concentrations (100 and 200 μM) of 2, 4-D to pretreat the roots of *Ae. variabilis No.1* to test CCN resistance. Statistical results revealed that there was no difference of CCN number in the roots between IAA pretreat and control (**Supplementary Figure 3**). These results indicate that IAA has no impact on the interaction between CCN and *Ae. variabilis No.1*. In addition, IAA is likely subjected to strict monitoring during the interactions of plants and nematodes.

## Author Contributions

HZ and MY designed the experiments. HZ and QH carried out sample collection, expression analysis, gene silencing, disease resistance assessment, data analysis, and wrote the manuscript. LL transformed transgenic tobacco. MZ analyzed transcriptome data. HL, GD, ZP, JL, QL, and FC assisted the experiments. All authors approved the final manuscript.

## Conflict of Interest Statement

The authors declare that the research was conducted in the absence of any commercial or financial relationships that could be construed as a potential conflict of interest.

## Footnotes

1 www.shimadzu.com.cn/

2 www.appliedbiosystems.com.cn/
