# Corrigendum: The *Tryptophan decarboxylase 1* Gene From *Aegilops variabilis No.1* Regulate the Resistance Against Cereal Cyst Nematode by Altering the Downstream Secondary Metabolite Contents Rather Than Auxin Synthesis

**DOI:** 10.3389/fpls.2019.01271

**Published:** 2019-10-10

**Authors:** Qiulan Huang, Lin Li, Minghui Zheng, Fang Chen, Hai Long, Guangbing Deng, Zhifen Pan, Junjun Liang, Qiao Li, Maoqun Yu, Haili Zhang

**Affiliations:** ^1^Chengdu Institute of Biology, Chinese Academy of Sciences, Chengdu, China; ^2^College of Life Sciences, Sichuan University, Chengdu, China; ^3^University of the Chinese Academy of Sciences, Beijing, China; ^4^School of Basic Medical Sciences, Zunyi Medical University, Zunyi, China

**Keywords:** cereal cyst nematode, Aegilops variabilis No.1, Tryptophan decarboxylase, secondary metabolite, indole acetic acid

In the published article, there was an error in affiliations 2 and 3. Instead of “University of the Chinese Academy of Sciences, Beijing, China” and “College of Life Sciences, Sichuan University, Chengdu, China,” it should be “College of Life Sciences, Sichuan University, Chengdu, China” and “University of the Chinese Academy of Sciences, Beijing, China.”

The authors apologize for this error and state that this does not change the scientific conclusions of the article in any way. The original article has been updated.

