# Supplementary material for: The Tryptophan decarboxylase 1 Gene From Aegilops variabilis No.1 Regulate the Resistance Against Cereal Cyst Nematode by Altering the Downstream Secondary Metabolite Contents Rather Than Auxin Synthesis
Source: Front Plant Sci. 2018 Sep 4;9:1297. doi: 10.3389/fpls.2018.01297 (PMC6132075; doi:10.3389/fpls.2018.01297)
Supplement: Supplementary file 1 [file Table_1.docx]

Supplementary Material

*Tryptophan decarboxylase 1* from *Ae.variabilis* *No.1* regulated resistance to cereal cyst nematode by altering secondary metabolite contents

Qiulan Huang^1,2,3^ , Lin Li^4^ , Minghui Zheng^4^ , Fang Chen^3^,Hai Long^1^ , Guangbing Deng^1^ , Zhifen Pan^1^, Junjun Liang^1^, Qiao Li^1^, Maoqun Yu^1 *^ , Haili Zhang^1*^

*** Correspondence:**

Corresponding Author: Maoqun Yu, Haili Zhang

yumaoqun@cib.ac.cn, zhanghl@cib.ac.cn

# Supplementary Table 1 Forward (F) and reverse (R) primers used for real-time quantitative PCR

| Primer Sequence From (5'-3') |  |
| --- | --- |
| GCTTGCTAATGGAGAATCTCAACA | real-time PCR primers for AeVTDC-1 |
| AATCCATGATACTGCTCGTGGTCT |  |
| GTGTGTTGATGAAGAACCTGAATAA | real-time PCR primers for AeVTDC-2 |
| CATCACCATCTTGTTGGTTGTGC |  |
| CGCCATCATTTCGCCATCC | real-time PCR primers for AeVNIT2 |
| TGCTTCGGTTCGGTCTTCA |  |
| TCTTCACGAACGAGGACTACG | real-time PCR primers for AeVAAO2 |
| GGACGCCTTGGAGGAGAG |  |
| CTACCCAAAGACGGAGTTCC | real-time PCR primers for AeVYUCCA |
| AGACGACGGAGATAGACCTC |  |
| CGATGGGCTACCTCCACCAG | real-time PCR primers for AeVSAUR15 |
| GATGGCGACGAAGTCCTCCT |  |
| AGGGCATCAACTTCGACCTC | real-time PCR primers for AeVCOMT |
| TAGCAGTTCTTGAGCAGCGT |  |
| GATGGTGGGCGTGTTTGT | real-time PCR primers for AeVASMT |
| AGCTGTCCGCTACCATGC |  |
| GCTGTGGATGGTATTGAGACTC | real-time PCR primers for NtTDC |
| TCATTACTCTGAGGTGGTCGTA |  |
| GCGGAGGTAGAAGAGGTTGT | real-time PCR primers for NtT5H |
| AGAGCCTGTTGATGGTTGGA |  |
| CGTCAAGTCCAGAATCCAAGAA | real-time PCR primers for NtASMT |
| CTCACTAACATGATGCCAAGGT |  |
| GGCACAGATCCAAGATTCAACA | real-time PCR primers for NtCOMT |
| TTCCACCACCAACATCAACAAT |  |
| TGTGGCACTGTTGAACTTGT | real-time PCR primers for NtYUCCA |
| AGCCCTTTCCTTGTAAATCCAA |  |
| CAATTCATGGCTCGGAAGGA | real-time PCR primers for NtSS |
| AACTTCAAGGTTTTGCTCTCG |  |
| CCAAGATTGACAGGCGTTCT | real-time PCR primers for NtEF1A |
| TGATGACACCAACAGCAACA |  |
| CCTCAAATCCATCGTCTT | real-time PCR primers for AeVT5H |
| TGTCATCGTCCACTCTAT |  |
| AACTACGGGTCGGTGAAGAA | real-time PCR primers for AevMES |
| GTCGGCAATCCTGAGAAGAAC |  |
| AAGGATCTGAAGCGTGGG | real-time PCR primers for AvEF1α |
| TGTGTGAGGTGTGGCAGTC |  |
| GTCATGCAAGCTGATGCC | vigs primers for AeTDC1 |
| TACGCCATCAAGATGGTGGC |  |
| ATGGGCAGCTTGGGCACCAAC | primers for AeVTDC1 ORF |
| TTAATCCATGATACTGCTCG |  |
| CTTTAGGATCAAGGCAAGTG | specific primers for screening AvTDC1 transgenic tobacco |
| GCACCATAAGTAAGTGTTGT |  |
| AACCGCTTGCTAATGGAGAATC | real-time PCR primers for TaTDC1 |
| TACTGCTCGTGGTCTTCTTGAT |  |
| CGCCTAAGTGACTCGTTGGA | real-time PCR primers for TaTDC2 |
| GCTACGGATGTGCTCTTGGA |  |

## Supplementary Figures


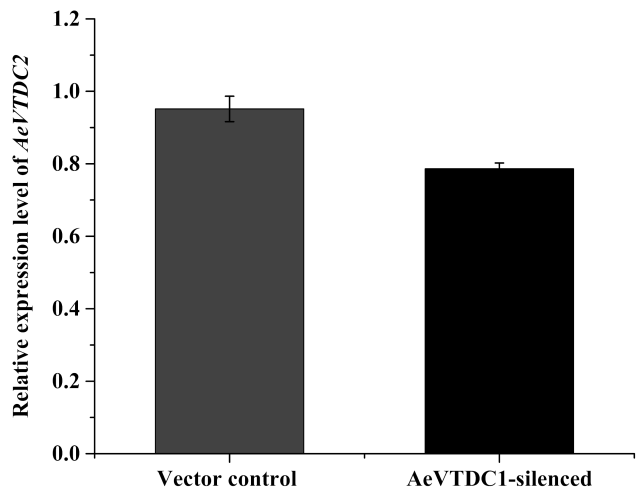


Supplementary Figure 1: The relative expression of *AeVTDC2* in roots of vector control and *AeVTDC1*-silenced plants. The results were normalized with the *AeVEF1α*.


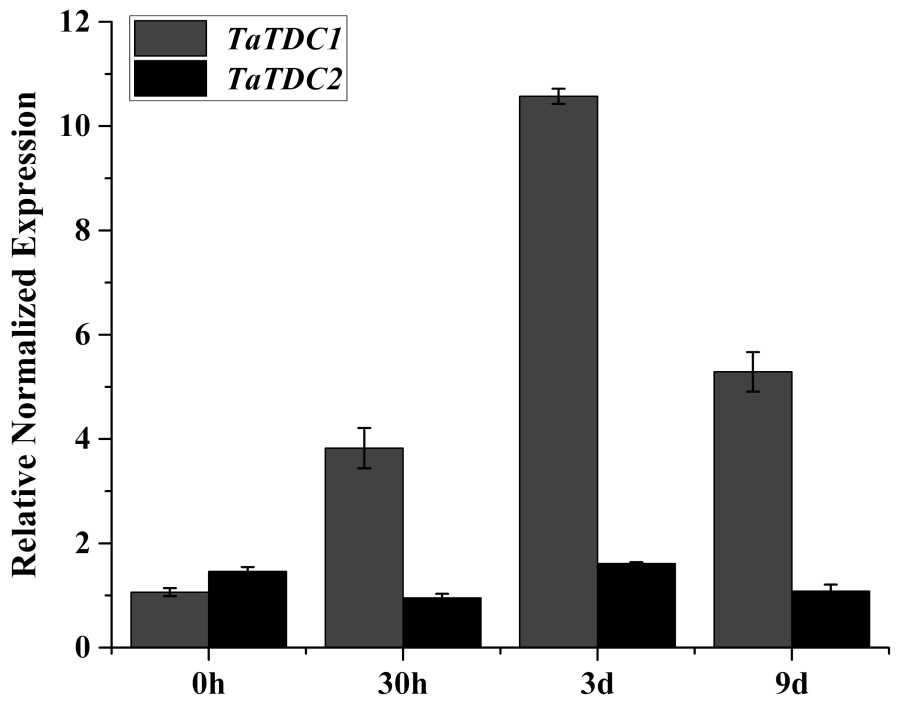


Supplementary Figure2: Expression of *TaTDC1* and *Ta TDC2* gene after CCN inoculation in wheat roots.

Two-leaf stage seedlings were used for CCN J2 inoculation. The roots tissues were respectively collected at 0 h, 30 h, 3 d and 9 d post inoculation for RNA extraction and QPCR. The relative expressions were normalized with the *TaEF1α*. Data represent the mean ± standard deviation of three replicate samples.





Supplementary Figure3. CCN number in the roots of IAA pretreat *Ae.variabilis No.1* and control.

Two-leaf stage seedlings of *Ae.variabilis No.1* were cultured in water contain 100μM /200μM 2,4-D for 24 h , the control plants were cultured in the water contain isopyknic 2,4-D alcohol solving liquid. These seedlings were grawn in the sterilized soil for one day and then inoculated CCN around the root-tips under 19℃, 300 J2s CCN per pot, each pot had four seedlings . The number of visible pink-stained nematodes present within the roots was counted under a light microscope. The data were means ± SE. No less than 15 plants were used and calculated in each treatment.
